# An Intelligent Optical Dissolved Oxygen Measurement Method Based on a Fluorescent Quenching Mechanism

**DOI:** 10.3390/s151229837

**Published:** 2015-12-09

**Authors:** Fengmei Li, Yaoguang Wei, Yingyi Chen, Daoliang Li, Xu Zhang

**Affiliations:** College of Information and Electrical Engineering, China Agricultural University, 17 Tsinghua East Road, Beijing 100083, China; lifm@cau.edu.cn (F.L.); chyingyi@126.com (Y.C.); dliangl@cau.edu.cn (D.L.); zhangxu_zx888@sina.com (X.Z.)

**Keywords:** dissolved oxygen, fluorescent quenching, intelligent compensation

## Abstract

Dissolved oxygen (DO) is a key factor that influences the healthy growth of fishes in aquaculture. The DO content changes with the aquatic environment and should therefore be monitored online. However, traditional measurement methods, such as iodometry and other chemical analysis methods, are not suitable for online monitoring. The Clark method is not stable enough for extended periods of monitoring. To solve these problems, this paper proposes an intelligent DO measurement method based on the fluorescence quenching mechanism. The measurement system is composed of fluorescent quenching detection, signal conditioning, intelligent processing, and power supply modules. The optical probe adopts the fluorescent quenching mechanism to detect the DO content and solves the problem, whereas traditional chemical methods are easily influenced by the environment. The optical probe contains a thermistor and dual excitation sources to isolate visible parasitic light and execute a compensation strategy. The intelligent processing module adopts the IEEE 1451.2 standard and realizes intelligent compensation. Experimental results show that the optical measurement method is stable, accurate, and suitable for online DO monitoring in aquaculture applications.

## 1. Introduction

Dissolved oxygen (DO) refers to the oxygen molecules dissolved in water and is essential to maintain human and animal life. Oxygen is an important analyte because of its key role in the life science, biotechnology, medicine, and aquaculture industries. The DO content in water is an indication of water quality, and careful control of oxygen levels is important in the self-purification processes of wastewater [1,2]. Water quality is closely related to the contaminants present in water, such as H_2_S, NO_2_, NH_4_^+^, and organic matter. Wastewater characteristics, including color, chemical oxygen demand (COD), and biological oxygen demand (BOD), specifically indicate the level of pollutants in industrial wastewater [3]. At the same time, DO plays a very important role in the health and growth of aquatic organisms [4,5]. A DO content of less than 2 mg/L for a certain number of hours causes the suffocation and death of aquatic organisms [6]. For humans, the DO content of drinking water should not be less than 6 mg/L. Consequently, the determination of oxygen concentrations is of high importance in the aquaculture industry and in daily life. However, monitoring the DO content with all its external influencing factors, such as temperature, pressure, and salinity, is difficult. To obtain an accurate DO content, the detection method should implement intelligent compensation. In general, three methods can be used to detect DO content: iodometric, electrochemical, and optical methods [7,8]. 

The iodometric method [9,10] is a popular and precise method of detecting the DO content in water. It is a fiducial method but has a complex detection process and cannot be used to detect the water quality online. This method is mainly used as a benchmark in the laboratory environment (off-line). The electrochemical method [11,12,13,14] uses electrodes to detect the current produced by redox reactions at the electrodes. This method can be classified as polarographic type or galvanic cell type based on the detection principle. The electrochemical method has a long history in the detection of DO content; the first so-called Clark polarographic method was designed by Clark of the YSI Company in 1956 [12]. In contrast to iodometry, the electrochemical method monitors DO content by the oxidation-reduction reaction that occurs between the electrode and DO molecules and consumes oxygen in the detection process. Given that instrumental drift is inevitable with the large number of factors that are involved in determining the detection result, electrochemical sensors require regular calibration and replacement. Optical DO sensors [15,16] are more attractive than the iodometry and electrochemical methods because they have a fast response time, do not consume oxygen, have a small drift over time, have the capability to withstand external disturbances, and require marginal calibration. The detection principle of optical DO sensors is based on fluorescent quenching, including fluorescent lifetime detection and fluorescent intensity detection. Intensity detection can be achieved through the photodiode, unlike lifetime, which should be detected based on the phase shift [17]. This study develops an intelligent optical measurement method based on the fluorescent quenching mechanism.

The aforementioned methods have some advantages and disadvantages that make them unsuitable to the aquaculture industry in China. First, the detection of DO content is difficult for the aquarist who must deal with numerous factors which influence the aquaculture industry. A comparison of the three methods shows that the electrochemical method is not a good choice because of its weak anti-interference properties. Second, the DO content is not constant, and insufficient concentration in natural water leads to the death of fishes. Thus, detecting DO content in real time is very important. However, iodometry method water samples must be tested in the laboratory, rendering this method unsuitable for monitoring organisms in actual production for this reason. Finally, traditional optical sensors have several disadvantages, including susceptibility to changes in external temperature, pressure, and salinity and attenuation of light source and drift because of the degradation or leaching of the dye. The influence off all these factors can be decreased by adding intelligent processing modules. The conventional optical DO sensor introduced from abroad is expensive and does not have high accuracy when used in the aquaculture industry. Thus, designing and developing an inexpensive and intelligent dedicated optical DO sensor is necessary.

This study proposes and develops an intelligent DO measurement method based on the fluorescent quenching mechanism. The sensor contains four modules: fluorescent quenching detection, signal conditioning, intelligent processing, and power supply modules. The sensor based on fluorescent quenching has several advantageous aspects: lower power consumption, smaller size, higher accuracy, and stronger anti-interference properties than iodometry or electrochemical sensors.

## 2. Materials and Methods

### 2.1. The Overall Design of Optical Dissolved Oxygen Sensor

**Figure 1:**
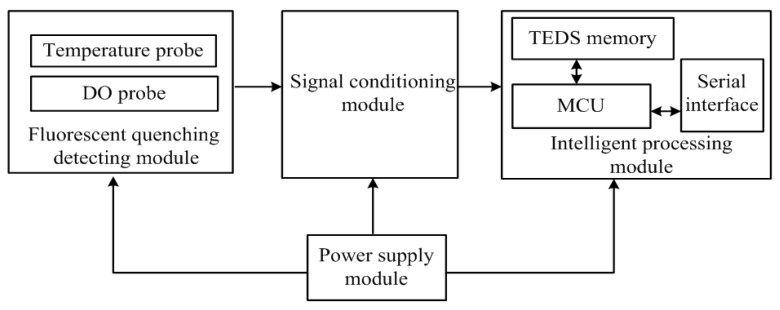
Overall design of the optical DO sensor.

Given the presence of unstable influencing factors, the sensor adopts an optical probe based on the quenching of fluorescence. Compared with the traditional DO sensor, the intelligent optical DO sensor proposed in this study has an enhanced probe structure and an additional intelligent processing module. These calibration parameters are stored in the transducer electronic data sheet (TEDS) memory. Figure 1 shows that the fluorescent quenching detection, signal conditioning, intelligent processing, and power supply modules are included in the intelligent sensor. 

The fluorescent quenching detection module contains a temperature probe and a DO probe. The temperature probe is responsible for collecting the water temperature signals, and the DO probe is responsible for collecting the DO signals. The original input signal can be converted into 0–2.5 V voltage signal by the signal conditioning circuits. The MSP430 microcontroller, which is the core of the intelligent processing module, is connected to the signal conditioning circuits, TEDS memory, and serial interface [18]. The collected data are fused through multi-probe data fusion technology, and the DO value obtained is transferred through compatible RS485 interface after being processed and analyzed by the microcontroller. The RS485 interface allows the microcontroller to communicate with the upper PC. The sensor is powered by an on-off power supply, which is also controlled by the MSP430 microcontroller. 

### 2.2. Design of the Fluorescent Quenching Detection Module

**Figure 2:**
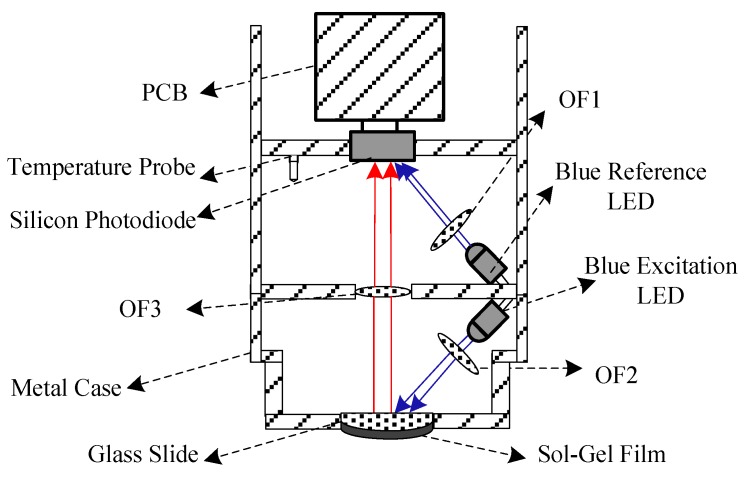
Schematic of the fluorescence quenching detection module.

The schematic of the fluorescent quenching detection module is shown in Figure 2. The probe has an approximate length of 16 cm and a diameter of 4 cm. The compact probe configuration is used for compatibility with the requirements of the aquaculture industry. As illustrated in Figure 2, the DO probe contains dual high-brightness blue LEDs, sol-gel film, glass slide, red optical filter, blue optical filter paper, and silicon photodiode. This module also includes a platinum resistance to monitor ambient temperature during measurements. The fluorescent intensity and temperature are processed in the software for temperature calibration.

The dual high-brightness blue LEDs (LA470-02) are modulated at the same frequency, so that the reference LED can be used to compensate the excitation LED because the light intensity loss of the blue LEDs are exactly the same. At the same frequency, the photodiode can reduce background radiation due to the ambient light in the measurement environment and avoid the excitation of any fluorescent material. In addition, the intensity of the LEDs are reduced to a low level at which the photobleaching phenomenon of the dye has a small probability of occurring [19]. The central wavelength of the blue excitation LED is about 465 nm, which is filtered by a blue bandpass filter paper to screen out the light of other wavelengths. The experimental results show that the blue light can induce the sensitive membrane to emit fluorescence at 650 nm. To reduce the influence of parasitic light, the probe is equipped with blue bandpass filter papers (OF1 and OF2) in front of the LEDs and red high-pass filter (OF3) in front of the silicon photodiode. A silicon photodiode (OPT 301) is used to receive the fluorescence emitted from the sol-gel film and blue light from the reference LED. The blue excitation LED and blue reference LED are separated on different sides of the red high-pass filter, which is beneficial in cutting off parasitic light and guaranteeing the accuracy of the optical signal detection.

The fluorescent sensing film is the most important part of the DO sensor and its performance significantly influences the accuracy, efficiency, and stability of the sensor. Researchers have conducted several studies on fluorescence indicators [20,21,22] and found that the most common fluorescent indicators contain metal porphyrin complexes, organic polycyclic aromatic hydrocarbons, and transition metal complexes [23]. Ru(bpy)_3_Cl_2_ has been chosen as the fluorescence indicator in this study because of its highly emissive metal-to-ligand charge-transfer state, long lifetime, and strong absorption in the blue-green region of the spectrum, which is compatible with the high-brightness blue LED [20]. The dye is entrapped in a porous and hydrophobic sol-gel film that is approximately 0.04 mm. The sol-gel film is mounted on the glass slide surface, which should be transparent for the excitation and luminescence to penetrate. The film should also be in the shape of an arc and maintain a stable size; the arc surface is designed to increase the area of contact and avoid surface bubble. The operating principle of the sensor is based on the fluorescent quenching mechanism. The fluorescent quenching process is described by the Stern-Volmer equation [24,25,26]:

(1) $$\frac{F_{0}}{F}=1+K_{sv}[Q]$$

where:

*F*_0_ denotes the fluorescence signal intensity of anaerobic water;
*F* denotes the fluorescence signal intensity of the water samples;
*K_SV_* denotes the Stern-Volmer constant; and
[*Q*] denotes the concentration of quencher.

As the equation shows, the degree of fluorescent quenching *F*_0_/*F* has a straight-line relationship with the concentration of the quencher [*Q*]. In this system, the quencher is the DO, so the DO content can be determined by using Equation (1).

### 2.3. Design of the Signal Conditioning Module

**Figure 3:**
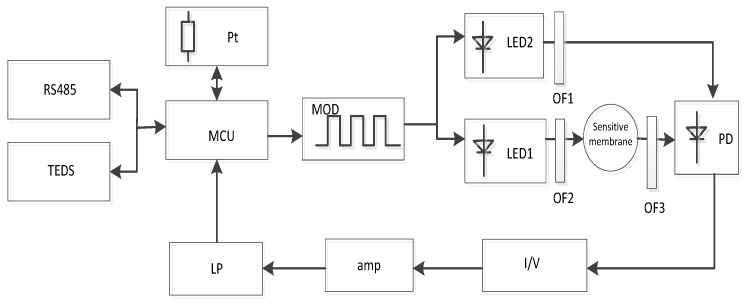
Schematic of the signal conditioning module.

The block diagram of the intensity measurement system is shown in Figure 3. The signal conditioning module is responsible for collecting and processing the information. The dual LEDs are modulated (MOD) into on-off at a frequency of 20 kHz. LED1 induces the sol-gel film to emit red fluorescence. At the same time, LED2 emits blue light as a contrast. The luminescence signal from the sensor dyes arrives time-delayed at the single photodiode (because of the luminescence lifetime of the dye), so that the reference and luminescence signals are detected in different time windows without interference. The fluorescence signal from the photodiode is converted to a voltage signal via an I/V switching circuit. The signal is then amplified (AMP) to obtain a voltage signal of 0–2.5 V and eliminate the higher harmonics of the signal. The voltage signal is filtered by a low pass (LP) filter and provides a voltage signal proportional to the measured intensity shift. Finally, the output voltage is divided by the voltage value based on the intensity in the anaerobic water to obtain F_0_/F, which has a linear correlation with the DO content. As presented in the Stern-Volmer equation, the DO content can be determined.

### 2.4. Design of the Intelligent Processing Module

With the rapid development of computers, communications, and networks, the DO sensor should be more intelligent, networked, and integrated [20]. To solve the problems of traditional optical sensors, the intelligent optical DO sensor proposed in this study is developed with automatic compensations, which are suitable for the online detection of aquaculture over long periods.

#### IEEE1451.2 Standard

**Figure 4:**
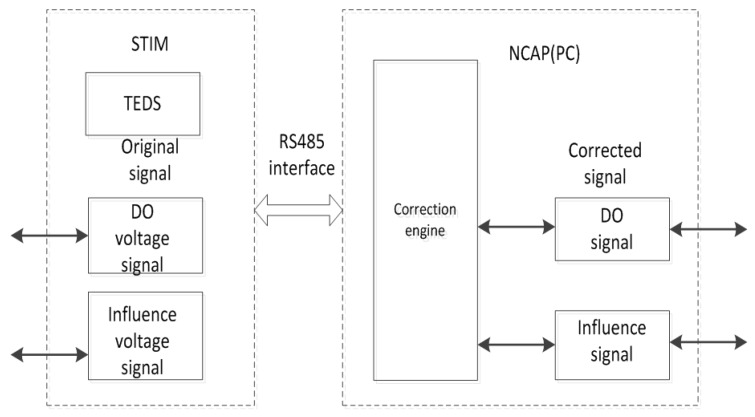
Model of data correction.

The data transfer is implemented using the protocols described in IEEE 1451.2 [27,28]. The intelligent sensor includes a smart transducer interface module (STIM) and network capable application processor (NCAP), and the sensor provides a general interface standard and a TEDS standard format for the intelligent sensor and the field bus. NCAP is connected to the STIM through an RS485 interface, as shown in Figure 4.

The calibration parameters are stored in the TEDS as shown in Figure 4. Through these parameters, the original signals can been converted into corrected signals, including DO, temperature, pressure, salinity, and luminous flux signals.

(2) $$f(X_{1},X_{2},...X_{n})=\sum_{i=0}^{D(1)}\sum_{j=0}^{D(2)}...\sum_{p=0}^{D(n)}C_{i,j...p}{\left[X_{1}-H_{1}\right]}^{i}{\left[X_{2}-H_{2}\right]}^{j}...{\left[X_{n}-H_{n}\right]}^{p}$$

In the equation, *X_n_* denotes the sensor output variables; *H_n_* denotes the revised output variables; *D_n_* denotes the order of output variables; and ***C_i,j,…,p_*** denotes the polynomial coefficient.

The calibration standard equation is shown in Figure 4. The relationship between the output variables and revised output variables, such as DO, temperature, pressure, salinity, and luminous flux signals are converted into a standard form. The sensor can directly determine accurate values when it uses the calibration parameters stored in the TEDS, which not only facilitates automatic recognition but also solves the sensor automatic correction problem. In the intelligent optical DO sensor, the total, channel, and calibration TEDS must be defined. In this study, the definition of total and channel TEDS makes the intelligent optical DO sensor more standardized, and that of the calibration TEDS may correct the original data at any time. These functions have significance for the maintenance and diagnosis of the sensors [29,30]. Thus, the intelligent optical DO sensor is called a “plug and play” device. 

## 3. Experiments and Discussions

### 3.1. Calibration and Validation

#### 3.1.1. Temperature Compensation of the Optical DO Sensor

The temperature dependence of the oxygen sensor has a number of contributions, including the temperature dependence of the fluorescence sensing film, light source, and the diffusion coefficient in the water. As discussed in Section 2.2, the light intensity related to temperature is measured by the probe, and the temperature effect is measured by the sensor based on the thermistor [31]. This study designs a correction model based on the temperature applicable to online DO monitoring. This model can perform temperature compensation to reduce the influence of temperature through the curve fitting algorithm.

**Figure 5:**
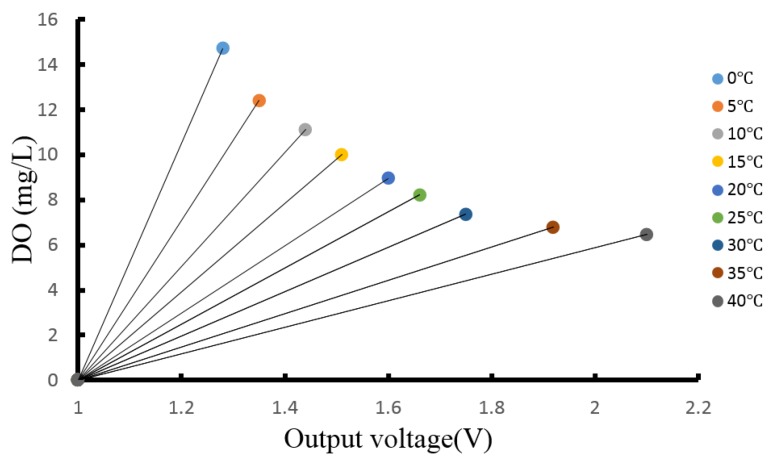
Line chart of the DO content and output voltage signal.

To eliminate the influence of temperature on sensitive film and light, anaerobic water and oxygen saturated solutions are prepared as standard solutions, which are heated with an integrated thermostatic magnetic blender (HWCL-1) from 0 °C to 40 °C in an off-light environment. The values are measured by a commercial DO meter (YSI 6150) in steps of 5 °C (0–40 °C), and the data between output signal and temperature obtained are displayed in the plot shown in Figure 5.

The relationship can be described by the following equations:

(3) $$\{\begin{array}{c} DO_{t1}=\beta_{1}+\beta_{t1}U\\ DO_{t2}=\beta_{2}+\beta_{t2}U\\ \vdots \\ DO_{tn}=\beta_{n}+\beta_{tn}U \end{array}$$

**Figure 6:**
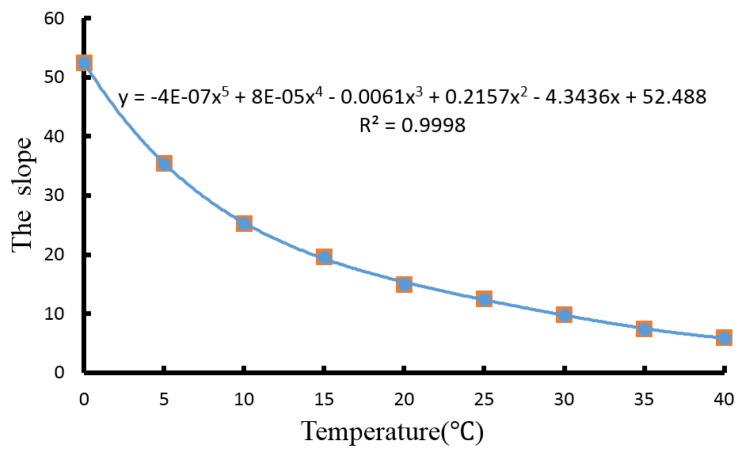
Fitting curve of the slope.

The slope is an important parameter to achieve the DO content, which changes with temperature. The relationship between the slope and temperature can be obtained based on the curve fit method in this study. As shown in Figure 6, the slope of the output voltage signal can be fitted by Equation (4):

(4) $$\beta_{t}=\alpha_{0}+\alpha_{1}T+\alpha_{2}T^{2}+\alpha_{3}T^{3}+\alpha_{4}T^{4}+\alpha_{5}T^{5}$$

In the equation, α_0_ = 52.488, α_1_ = −4.3436, α_2_ = 0.2157, α_3_ = −0.0061, α_4_ = 0.00008, α_5_ = −0.0000004, and R^2^ = 0.9998, as seen in Figure 6, which denotes a very good linear fitting. The DO content can be obtained at any temperature, taking Equation (4) into Equation (3), and Equation (5) can then be achieved.

(5) $$DO_{t}=\beta +\alpha_{0}U+\alpha_{1}UT+\alpha_{2}UT^{2}+\alpha_{3}UT^{3}+\alpha_{4}UT^{4}+\alpha_{5}UT^{5}$$

The calibration standard Equation (2) in the TEDS calibration can be adapted as follows:

(6) $$DO_{t}=\sum_{i=0}^{1}\sum_{j=0}^{5}C_{ij}{\left[U-H_{1}\right]}^{i}\cdot {\left[T-H_{2}\right]}^{j}$$

If H_1_ = 0 and H_2_ = 0, then Equation (6) can be presented by Equation (7):

(7) $$\begin{array}{l} DO_{t}=C_{00}+C_{01}T+C_{02}T^{2}+C_{03}T^{3}+C_{04}T^{4}+C_{05}T^{5}\\ +C_{10}U+C_{11}UT+C_{12}UT^{2}+C_{13}UT^{3}+C_{14}UT^{4}+C_{15}UT^{5} \end{array}$$

The calibration values are consistent with the actual temperature and output voltage. The DO value must be corrected according to the real-time temperature. Taking C_00_ = β, C_00_ − C_05_ = 0, C_10_ = α_0_, C_11_ = α_1_, C_12_ = α_2_, C_13_ = α_3_, C_14_ = α_4_, and C_15_ = α_5_ into Equation (7) and detecting the temperature and output voltage, the accurate DO value can be determined in the 0–40 °C range (*i.e.*, the range of the sensor). The temperature probe in this sensor can track the temperature response of the DO sensor in real time, thereby achieving temperature compensation in an application where frequent short-term changes in temperature occur. The calibration curve based on intensity measurement changes because of the impactsof typical drift sources, including the leaching of the indicator, LED output, and ageing of the sol-gel matrix. Thus, the sensor requires recalibration after a certain period, but the frequency achieves much more progress than the electrochemical sensor. In addition, the temperature compensation should be processed in the shade to avoid the influence of daylight and background fluorescence.

#### 3.1.2. Pressure Compensation of the Optical DO Sensor

Given that the DO value increases when the sol-gel film is moderately affected by pressure, the pressure affects the accuracy of the measurement. Pressure compensation is needed in the measurement of oxygen, and the correction formula [32] is as follows:

(8) $$DO^{\prime }=C_{S}*\frac{P}{101.3}$$

In the equation, *DO*′ denotes the DO in the pressure of P; *C_S_* denotes the normal atmospheric pressure value; and *P* denotes the actual atmospheric pressure value.

**Figure 7:**
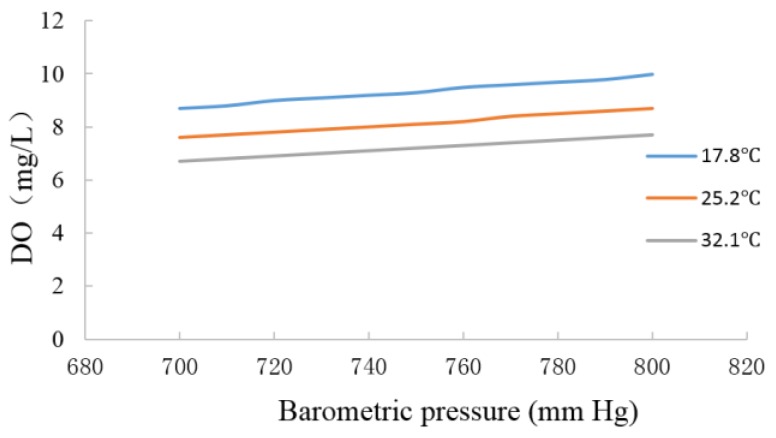
Pressure compensation test.

*C_S_* can be obtained through the national standard table at different temperatures. At different temperature, the aqueous solutions are subjected to different pressures. As mapped in Figure 7, the experiment shows that the optical DO sensor introduced in this study can effectively eliminate the effect of pressure and that the pressure compensation has a good effect.

#### 3.1.3. Salinity Compensation of the Optical DO Sensor

Given that the solubility of oxygen in water decreases as the salt content increases, the DO must be compensated according to the salinity. When the salinity (*i.e.*, expressed as the total salinity) is below 35 ppt, the correction formula [32] is given by:

(9) $$DO^{\prime \prime }=C_{S}-n\Delta C_{S}$$

In the equation, *DO*″ denotes the DO value at a salinity of n; *C_S_* denotes the DO value in pure water; *n* denotes the salinity value; and Δ*C_S_* denotes the reduction of DO according to salinity (1 ppt).

**Figure 8:**
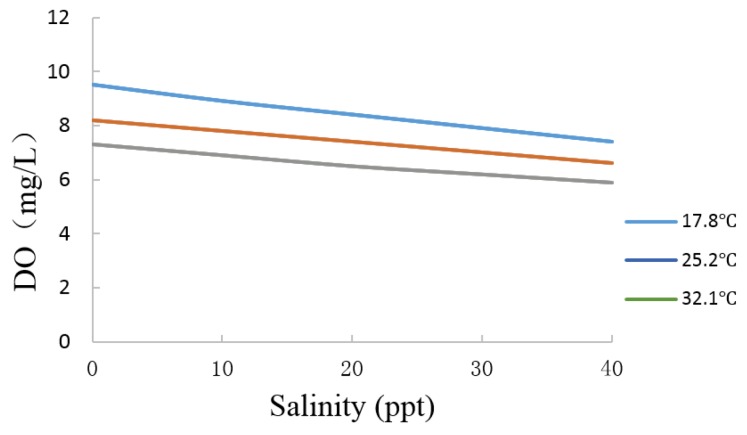
Salinity compensation test.

*C_S_* and Δ*C_S_* can be obtained through the national standard table at different temperatures. The aqueous solution is measured using KCl, NH_4_^+^, and CO_3_^2−^ as ions in the experiment. The ion density of the measurement solutions is between 0 and 40 ppt. The DO values measured separately are shown on the basis of the experimental data in Figure 8. The analysis of the data shows that the DO values have certain effects when ionic strength regulators are added. The experiment shows that the optical sensor satisfies the salinity compensation in the aquatic environment. The salinity compensation parameters are also stored in the calibration TEDS.

#### 3.1.4. Excitation LED Compensation 

The experimental results show that different intensities of the excitation LED have a large influence on the fluorescence intensity of the sensitive film. The intelligent processing module also allows for the adjustment of the calibration parameters to correct the excitation blue LED at the time of measurement (*i.e.*, if this differs from the excitation at the time of calibration). The luminous flux attenuation curve of the blue LED shows a similar exponential form at 100 °C. When the service time reaches about 10,000 h, the luminous flux of the blue LED (L470-02) drops to about 70% of the original luminous flux. Thus, when the LEDs work beyond 10,000 h, the sensor probe must be replaced. Before replacement, illuminant compensation is required to guarantee the accuracy of the DO sensor. The experimental results show that this sensor can effectively compensate the effect of the light source caused by the instability or aging of the light source. The compensation formula is shown in the following equation, and the calibration parameters are also stored in the TEDS. When the intensity of the reference blue LED changes, Equation (8) is used to correct the intensity value of fluorescence quenched by oxygen:

(10) $$E_{\delta }=E_{\gamma }\cdot \frac{e_{\alpha }}{e_{\beta }}\;(0.7\;\leq \;\frac{e_{\beta }}{e_{\alpha }}\;\leq \;1)$$

In the equation, *e_α_* denotes the original intensity value of the reference LED; *e_β_* denotes the measured intensity value of the reference LED; *E_γ_* denotes the measured intensity value of the quenched fluorescence; and *E_δ_* denotes the corrected intensity value of the quenched fluorescence. 

The long-term stability of the sensor in aquaculture tank water has been continuously studied for over 12 months. With the passage of time, the sol-gel matrix is prone to losing its original features, which may cause drifts in calibration, leaching, and bleaching of the dye. As described above, if the intensity of the fluorescence declines (*i.e.*, after about 12 months), then the sensor probe should be replaced rather than the whole sensor device with the separate structure.

### 3.2. Analysis of the Performance of the Sensor

To verify the performance of the sensor, the optical DO sensor is tested in terms of our aspects: accuracy, stability, precision, repeatability, rapidity. Its superior performance proves that the intelligent optical DO sensor can be used to monitor the DO content in the aquaculture industry. The experimental processes are shown below.

#### 3.2.1. Accuracy Test 

In the laboratory, the room temperature is about 25 °C, and three groups of standard aqueous solutions are measured: 5.02, 9.98, and 18.03 mg/L. The defined oxygen contents are adjusted by mixing the oxygen and nitrogen with mass flow controllers. The sample contents are checked using a commercial DO meter. Compared with reference values, the experimental data in Table 1 show that the relative error is less than ±2%, the measurement error is less than 0.2 mg/L, and the resolution of the optical DO sensor is 0.01 mg/L within the range of 0–20 mg/L, which conforms to the accuracy requirements. At the same time, the temperature error is less than 0.5 °C, which shows that the temperature probe is accurate.

**Table 1:** Accuracy test.

Samples	Measurements								AVG	Absolute Error	Relative Error
	1	2	3	4	5	6	7	8			
5.02	4.86	4.79	4.89	4.93	4.89	4.91	5.09	4.98	4.92	0.1	1.99%
9.98	10.12	10.09	10.21	9.89	10.06	9.91	9.89	10.13	10.04	0.06	0.60%
18.03	17.85	17.83	17.91	17.87	17.96	17.87	17.93	18.03	17.91	0.12	0.67%

#### 3.2.2. Stability Test

**Figure 9:**
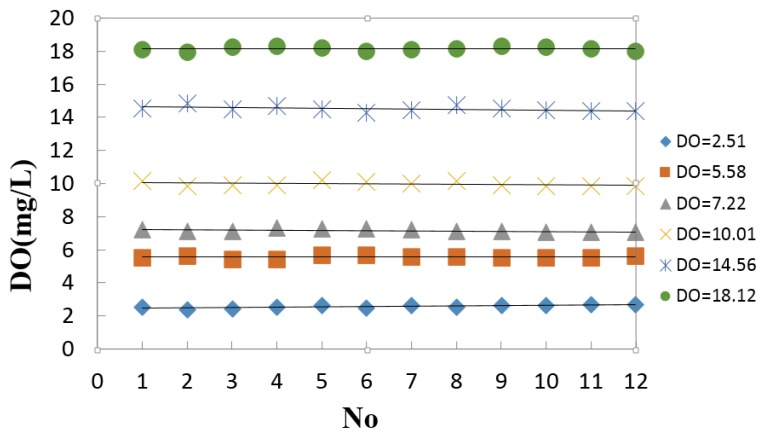
Stability test.

The response of the optical sensor appears to be stable within the measurement error, which meets the specified stability requirement of 0.2 mg/L. In the laboratory, the DO content is measured within 12 h in steps of 1 h. The standard aqueous solution is adjusted by mixing the oxygen and nitrogen with mass flow controllers. The experimental results are 2.51, 5.58, 7.22, 10.01, 14.56, and 18.12 mg/L. As shown in Figure 9, the largest error is less than 0.02 mg/L, which meets the requirements of the optical dissolved oxygen sensor.

#### 3.2.3. Precision Test

**Figure 10:**
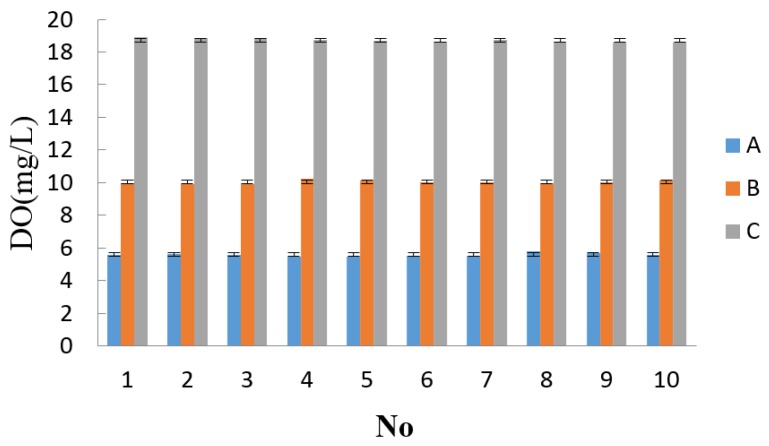
Precision test.

Precision refers to the discrete degree of the values that are repeatedly measured by the same method under the same experimental conditions for the same sample. Generally, the relative standard deviation (RSD) is used to explain the sensor precision. The RSD indicates that the results are more concentrated. The standard solution contains Na_2_SO_3_, and this sample is continuously measured ten times. The measurement results are shown in Table 2. The micro-controller calculates the RSD every ten measurements, indicating that the precision of this group of data is very good when the RSD is less than 2%. The micro-controller inputs these data into the internal memory. As shown in Figure 10, the results show that the sensor has a high precision.

**Table 2:** Precision test.

Sample	1	2	3	4	5	6	7	8	9	10	AVG Average average	RSD
A	5.56	5.62	5.60	5.44	5.49	5.51	5.50	5.82	5.71	5.60	5.59	1.97%
B	9.96	9.91	9.89	10.22	10.15	10.04	10.00	9.98	10.02	10.15	10.03	1.10%
C	18.90	18.85	18.86	18.74	18.69	18.56	18.74	18.63	18.60	18.59	18.73	0.64%

#### 3.2.4. Repeatability Test

Na_2_SO_3_ is added into the aqueous solutions to achieve the sample solutions (*i.e.*, 2.51, 9.24, and 12.57 mg/L). The sensor is used to measure the DO values of the sample solutions. The data are recorded every hour and continuously measured ten times. Table 3 shows that the relative error is less than 2%. The result of the data analysis indicate that the repeatability meets the requirements of the optical DO sensor.

**Table 3:** Stability test.

Samples	Measurement Results										Relative Error
	1	2	3	4	5	6	7	8	9	10	
2.51	2.54	2.5	2.44	2.56	2.62	2.48	2.63	2.55	2.59	2.63	1.59%
9.24	9.22	9.19	9.16	9.13	9.33	9.25	9.23	9.22	9.16	9.13	0.43%
12.51	12.49	12.44	12.45	12.44	12.67	12.71	12.58	12.55	12.49	12.51	0.16%

#### 3.2.5. Speed Test

**Figure 11:**
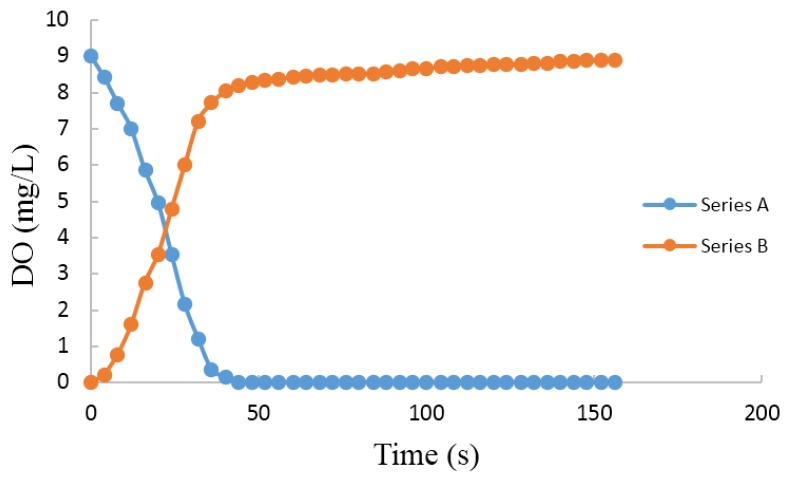
Speed Test.

Two cups of water are prepared in laboratory. 5% Na_2_SO_3_ and a suitable amount of CoCl_2_ are added to one of them. They are placed in the air for two days to ensure that both temperatures are same, which can eliminate the influence of temperature on the measurement results. Series A indicates the change of DO content from tap water to anaerobic water, and the Series B line indicates the change from anaerobic water to tap water. From Figure 11, we can see that the response time is less than 3 min. After 1 min, the Series A is nearly 0 mg/L. However, the response time of Series B is about 3 min. The reason of this phenomenon is that the process of fluorescence quenching must achieve dynamic balance. Series B should take some time to react, but it doesn’t need time in anaerobic water. The data indicates that detection response speed meets the requirements of the optical DO sensor.

#### 3.2.6. On-Site Verification Test

**Figure 12:**
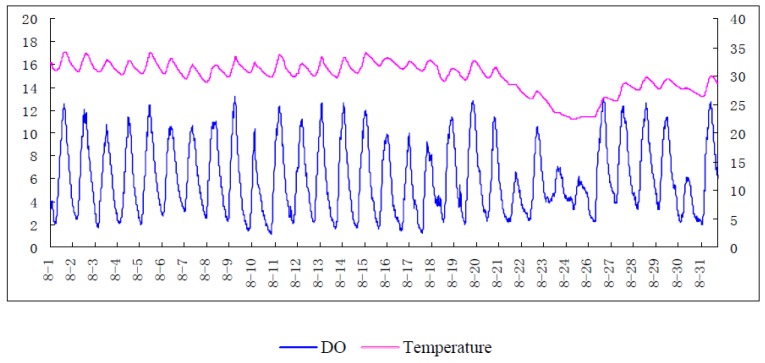
On-site Test.

Figure 12 is the real-time graph of dissolved oxygen changes in aquaculture ponds. After sunrise, phytoplankton produces photosynthesis, which releases a large amount of oxygen. The increase of oxygen is more than the consumption, so the DO content gradually increases. Over a period of time, at the peak of photosynthesis, DO content is at a maximum. After sunset, the phytoplankton undergo respiration which consumes oxygen instead of photosynthesis. In addition, with the respiration of aquatic animals, DO content decreases rapidly. After all night, the accumulation is reduced to a minimum at dawn. These changes perfectly accord with the test data, which proved that the developed DO sensor has a reliable performance. In contrast to change rates of oxygen consumed by organisms, the temperature has little influence on DO content in the on-site test.

## 4. Conclusions

To detect the dissolved oxygen content exactly in aquaculture industry applications, an intelligent optical dissolved oxygen measurement method based on a fluorescent quenching mechanism has been designed in this paper. The advantages of the unique sensor head based on fluorescent quenching have been highlighted and intensity measuring circuits and intelligent processing have been described. With the optical dissolved oxygen sensor probe in the paper, the detection of dissolved oxygen became more accurate and interference-free. In view of the influence of temperature, pressure, salinity and blue excitation LED on measurement, this paper has designed an intelligent processing module. The accurate DO content can be achieved by the intelligent optical DO sensor with the correction coefficients stored in the calibration TEDS. The experiments have shown that the sensor performance can meet the specifications for the monitoring DO content in aquaculture industry, such as low-power consumption, fast response, ability to detect online, high accuracy and strong stability, which will enable reliable operation for periods of months at the very least. In addition, the use of an optical measurement method and the intelligent calibration technique could be applicable in other medical and environmental monitoring areas.
